# What is a university education?

**Published:** 2018

**Authors:** HR Ahmad

**Affiliations:** Professor HR Ahmad MD PhD Bochum, FCPS. Aga Khan University, Karachi 74800, Pakistan

What are three functions of a university? These are knowledge production, dissemination and application through the means of research, learning and public service. The solid base of undergraduate education forms the foundation for research and graduate education at the university level. The research based education does not discriminate research from teaching. It is rather an integral part of knowledge production and dissemination through the scientific spirit. It is the driving spirit of objective inquiry. It is required throughout the entire educational process. It is a crucial part of quality education as well as indispensible factor for the evolution of modern scientific and universal cultural climate. Therefore, the scientific spirit should be treated as the creative energy for education at every step of educational process.

Since science deals with the relative truth, the established working model of a mechanism can be challenged and replaced by new ones. In this way science has become the lighthouse of progress for the human race. Since science deals with the testing of imagination by experimentation, the dearest imagination can stand against bitter data to test the critical thinking and the intellectual honesty. It may be inferred that research based education nurtures the scientific spirit as the fuel for the lighthouse that should never dim. To promote scientific spirit driven research should thus be the ultimate goal of a university enabling the critical experimental intellect of mentors and mentees to drive the generation cycle of an institution.

